# Supplementary material for: Quantitative single-cell proteomics as a tool to characterize cellular hierarchies
Source: Nat Commun. 2021 Jun 7;12:3341. doi: 10.1038/s41467-021-23667-y (PMC8185083; doi:10.1038/s41467-021-23667-y)
Supplement: Supplementary file 1 — Supplementary Information [file 41467_2021_23667_MOESM1_ESM.pdf]

Supplementary Information

Quantitative Single-Cell Proteomics as a Tool to Characterize Cellular Hierarchies

Schoof, Furtwängler et al.

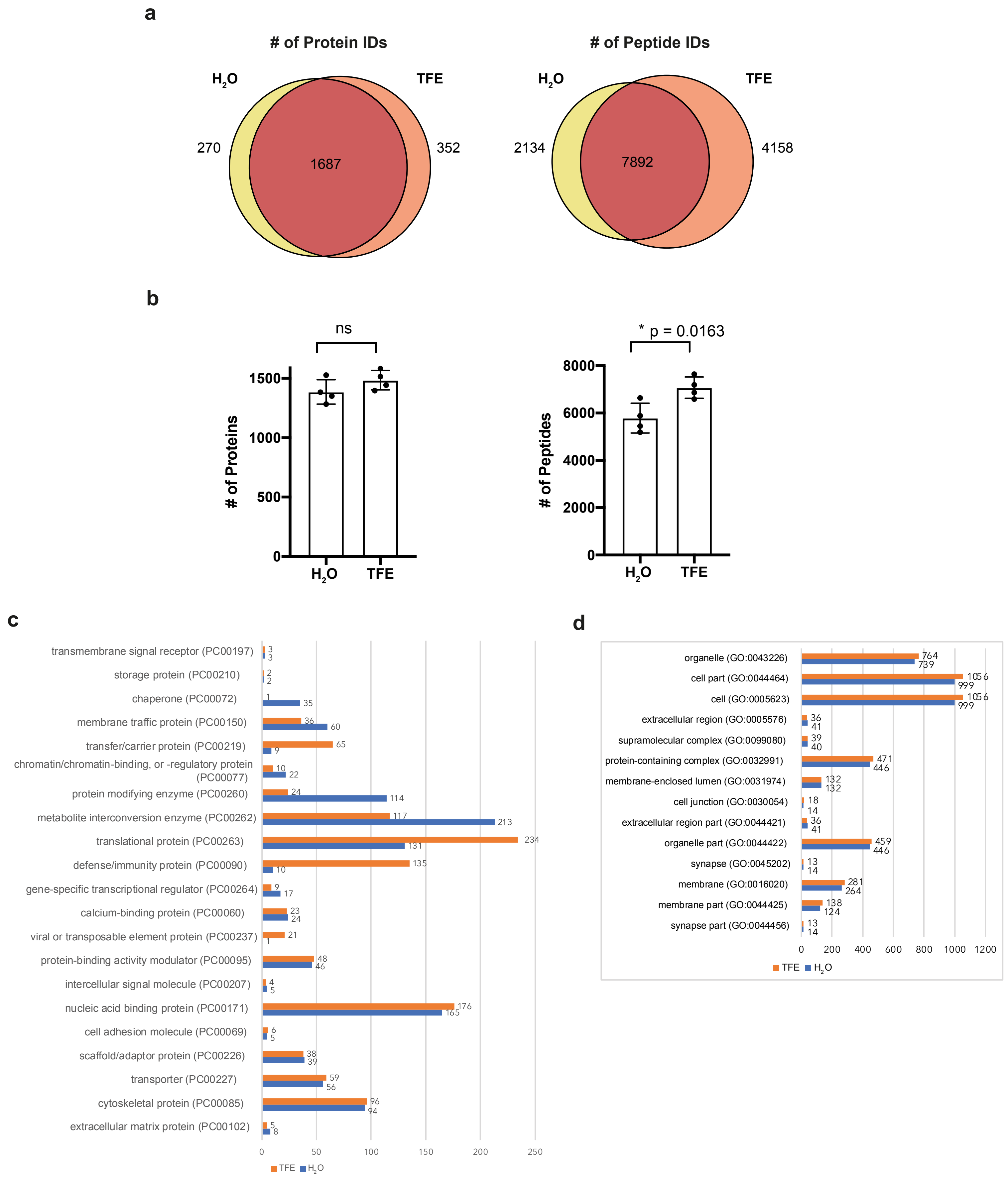

Supplementary Figure 1. Overview of protein and peptide identifications when using LC-MS grade water (H_2O) as lysis buffer for scMS experiments versus 20% trifluoroethanol (TFE), as measured in quadruplicate. (**a**) Venn diagram showing overlap of proteins and peptides identified for the two lysis buffers. (**b**) Barplots of the total number of peptides and proteins identified in each sample (n=4) for the two lysis buffers. P-values were calculated using an unpaired, two-tailed t-test and bars and error bars indicate mean values with SD. (**c**) & (**d**) PantherDB analysis of protein classes and cellular compartments represented by proteins detected with 20%TFE (n=1,873) and water (n=1,791). Source data are provided as a Source Data file.

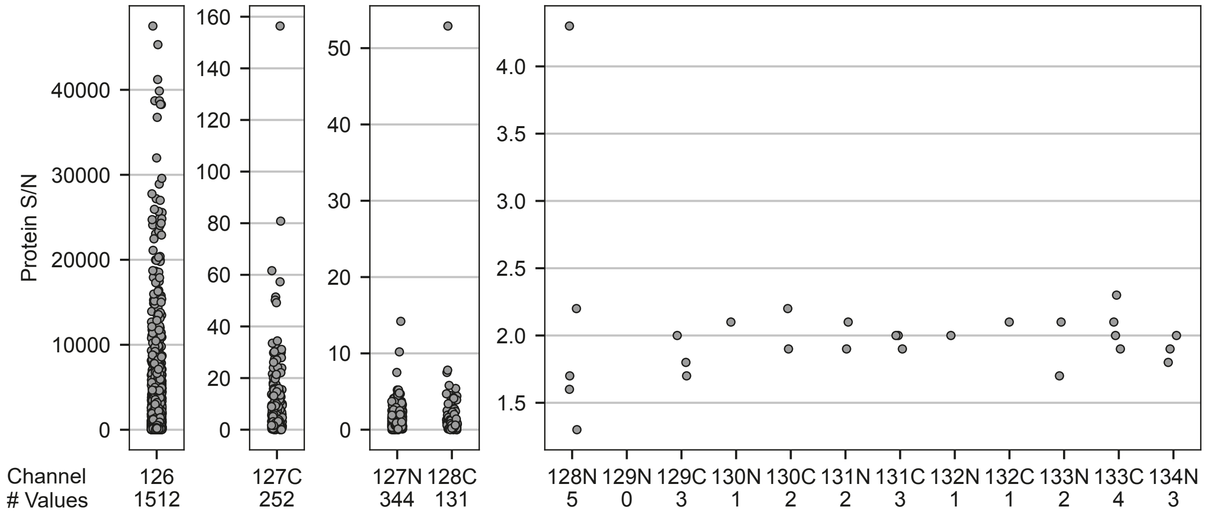

Supplementary Figure 2. Isotopic impurities of the 126 booster channel. The 1:1:1 booster was measured using the 'high' method, without the addition of other channels. Non-missing signal-to-noise (S/N) values on protein level in each TMT channel are plotted. Channels are grouped by signal intensity. Source data are provided as a Source Data file.

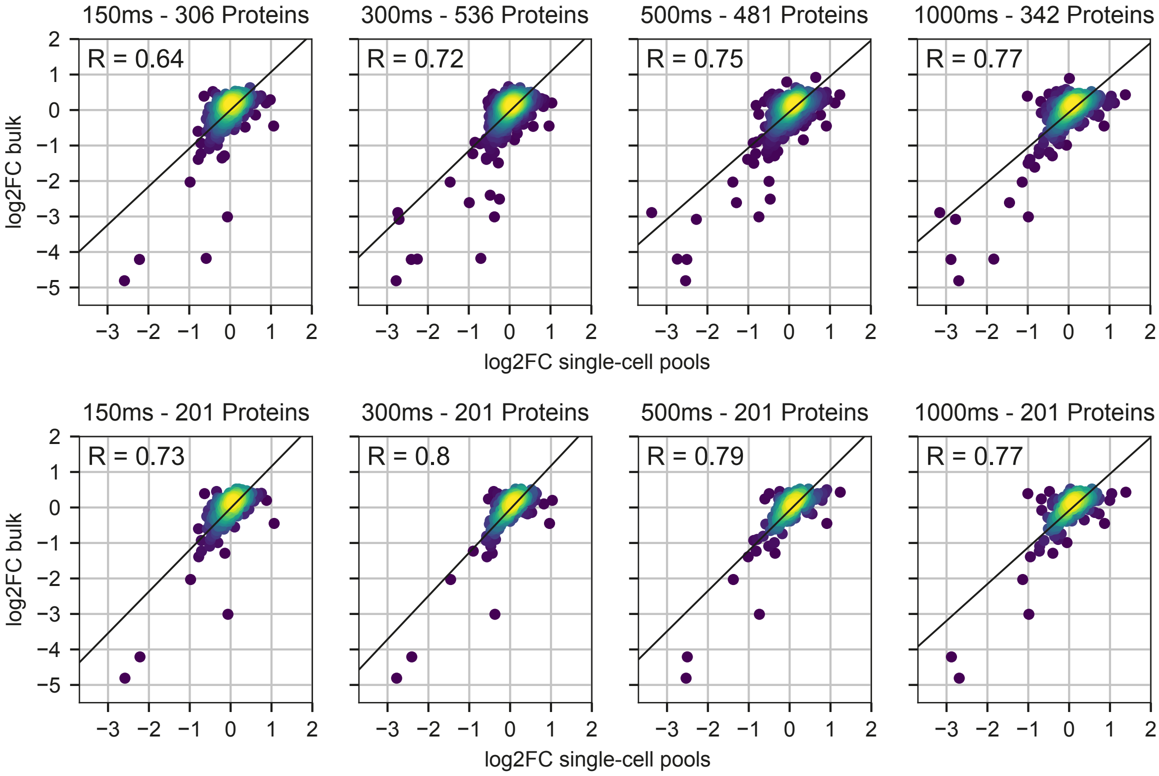

Supplementary Figure 3. Quantitative accuracy compared to MS3 bulk data. Pearson correlation of fold changes (log2FC) between LSC and blast in 'single-cell' samples and MS3 bulk data. Top: For each method, only proteins without missing values were considered. Bottom: Only proteins overlapping between all methods in top were considered. Source data are provided as a Source Data file.

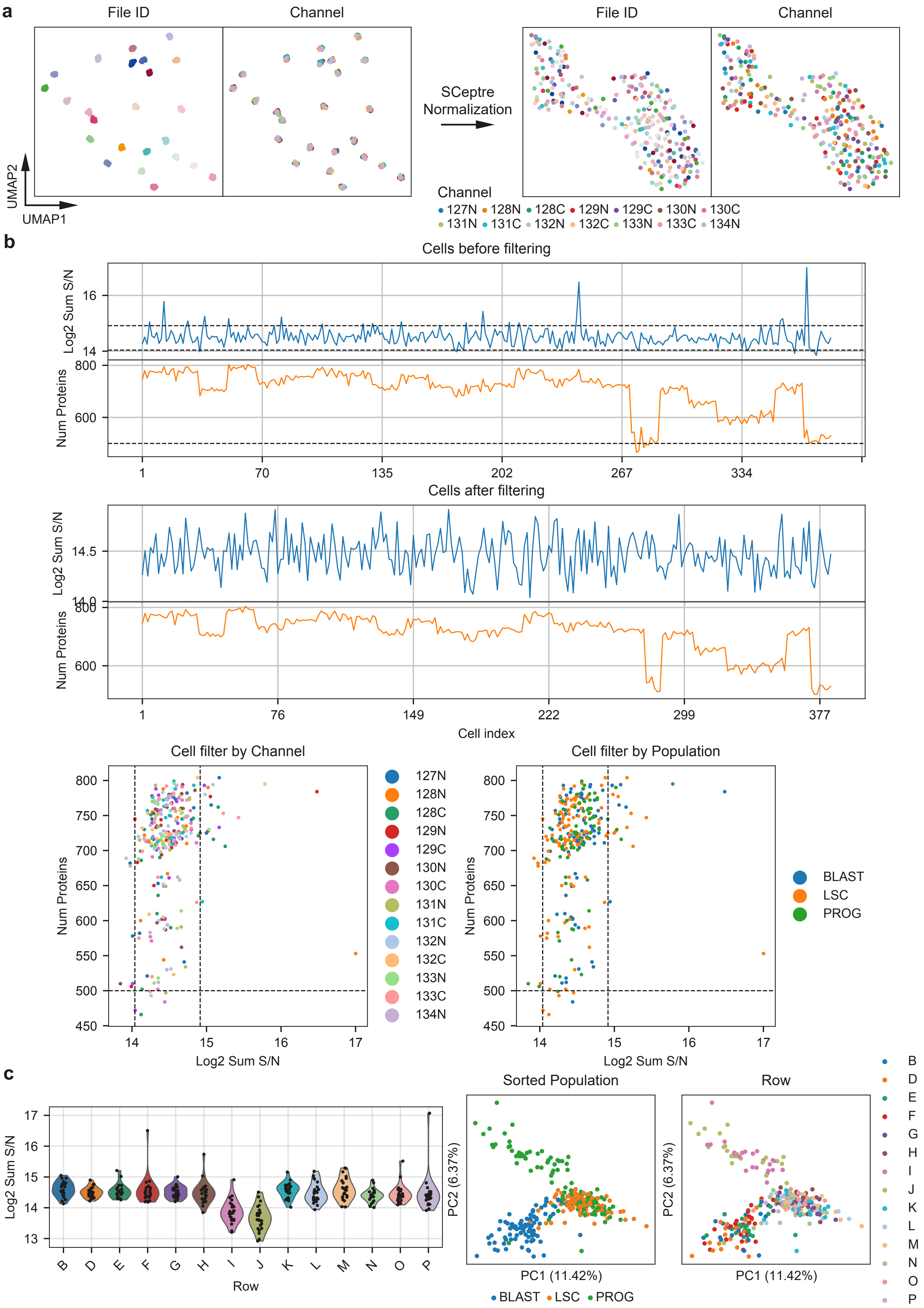

Supplementary Figure 4. SCeptre workflow of 'high' dataset. (**a**) Effect of SCeptre normalization. (**b**) Cell filtering based on Log2 Sum signal-to-noise (S/N) and the number of proteins per cell. BLAST = blasts, PROG = progenitors, LSC = leukemia stem cells (**c**) Batch effect in rows I and J detected by SCeptre. Source data are provided as a Source Data file.

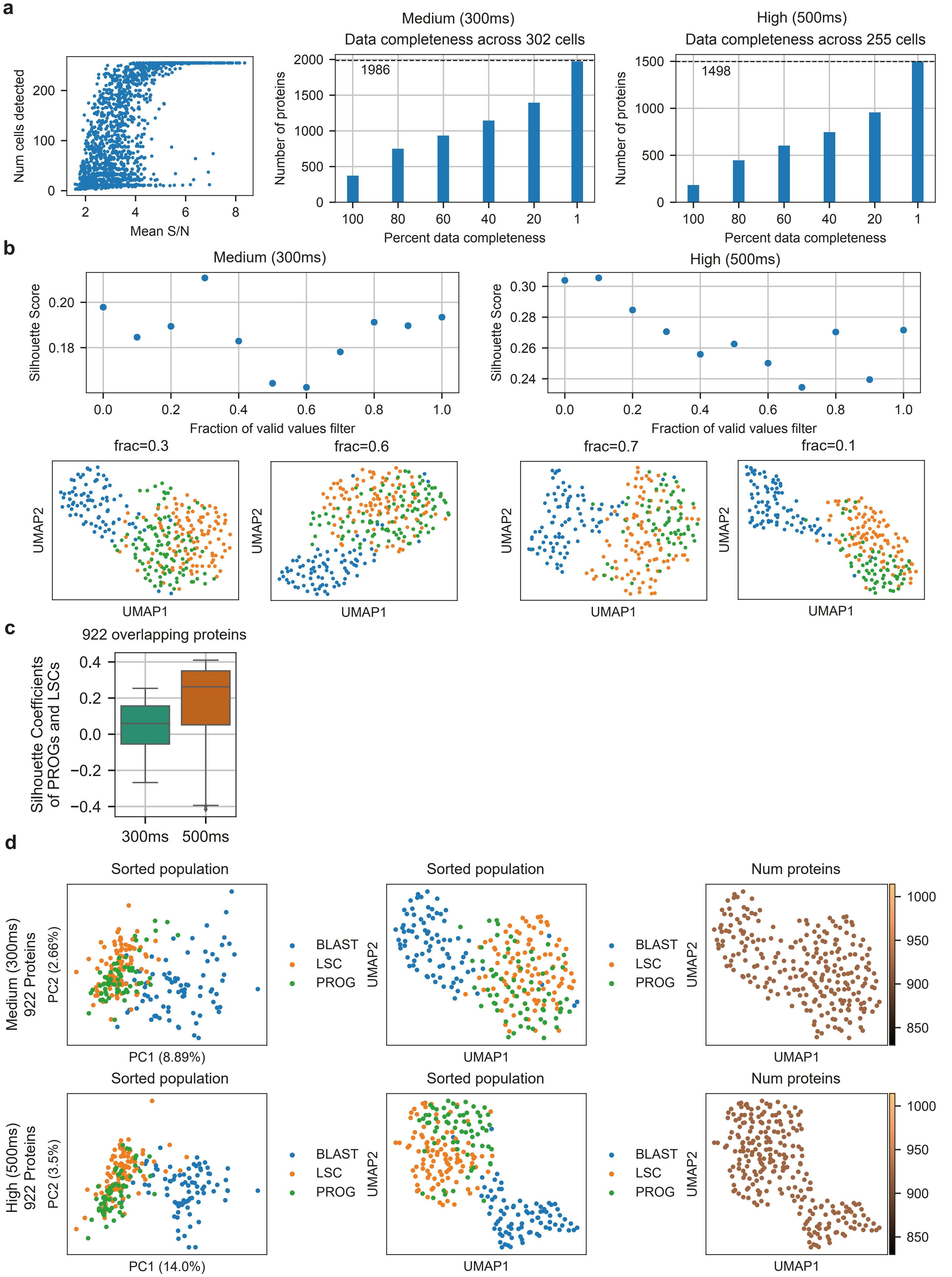

Supplementary Figure 5. Comparison between standard and high dataset. (**a**) Left: Proteins of 'high' dataset plotted with their coverage vs. their mean signal-to-noise (S/N) across cells. Right: Data completeness of 'medium' and 'high' dataset of 1986 proteins across 302 cells and 1498 proteins across 255 cells respectively. (**b**) Influence of coverage filter applied on proteins before imputation on the cell separation. (**c**) Silhouette coefficients with progenitors and LSCs when only 922 overlapping proteins and down-sampled cells were used (n=166 cells for both methods). Boxplot shows median, 0.25 and 0.75 quantile, and whiskers extend to points within 1.5 interquartile range of lower and upper quartile. (**d**) Comparison of embeddings when only 922 overlapping proteins and down-sampled 249 cells were used. BLAST = blasts, PROG = progenitors, LSC = leukemia stem cells. Source data are provided as a Source Data file.

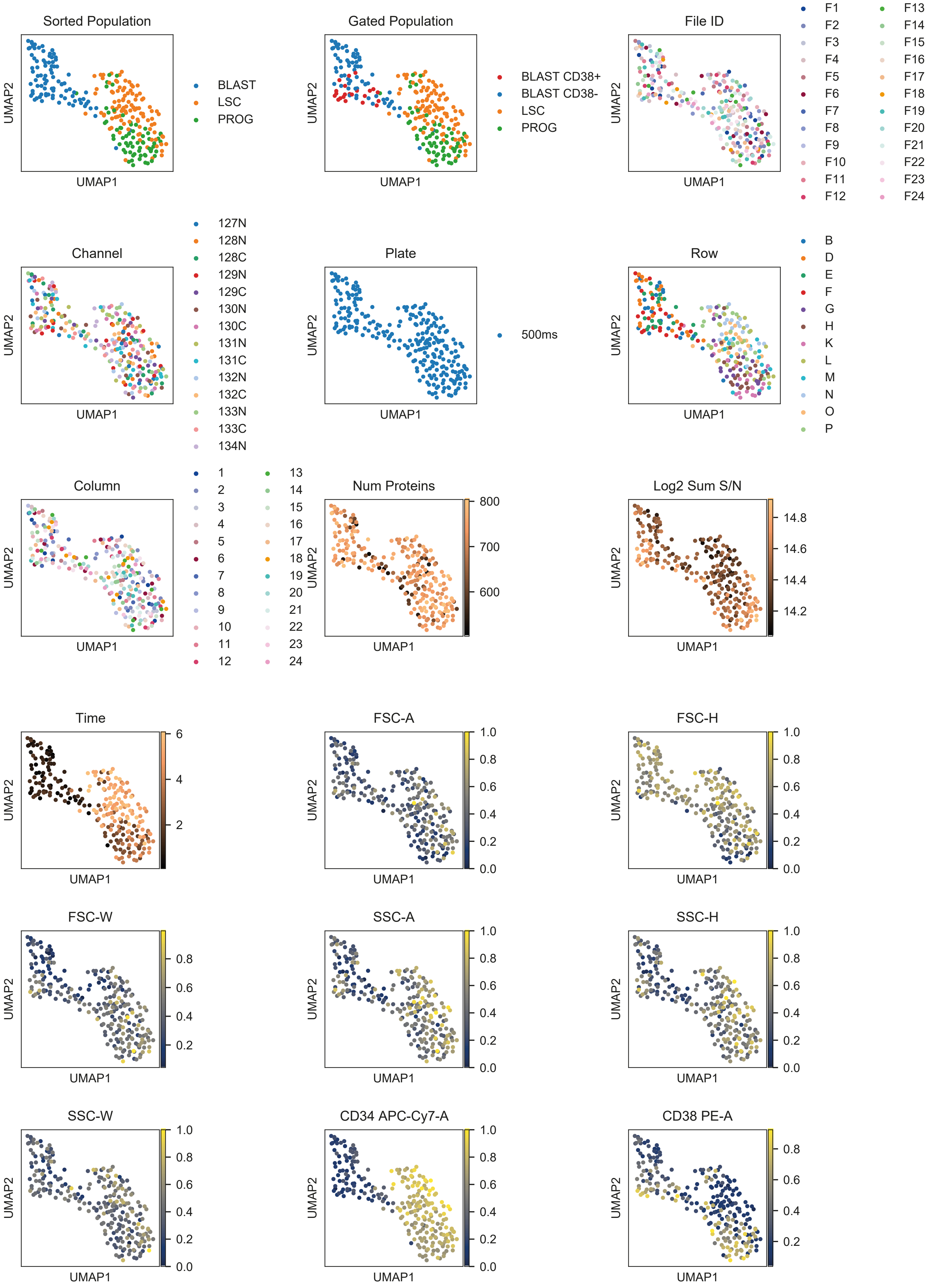

Supplementary Figure 6. UMAP with annotations of 'high' dataset. UMAP embedding of cells using scMS data, overlaid with annotations from the FACS sort and sample processing. BLAST = blasts, PROG = progenitors, LSC = leukemia stem cells. Source data are provided as a Source Data file.

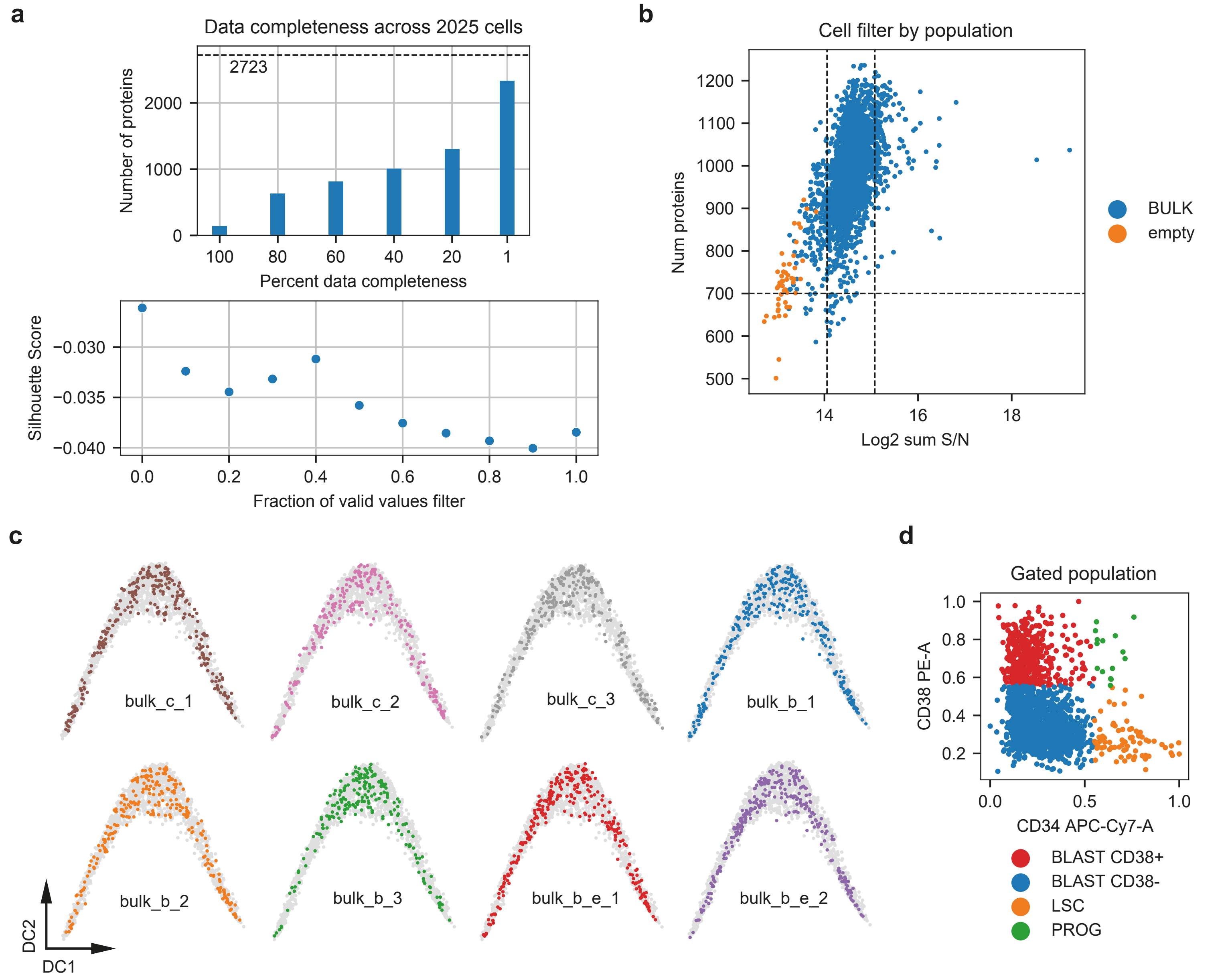

Supplementary Figure 7. Quality control of 'bulk' dataset. (**a**) Top: Data completeness of 2723 proteins across 2025 cells after filtering. Bottom: All 2,723 proteins were retained for imputation and embedding, as the silhouette score, based on the gated populations, was the highest in this case. Silhouette score was calculated from n=2025 cells. (**b**) Successful removal of all empty wells based on the summed signal intensity. (**c**) Successful integration of all 'bulk' plates, irrespective of booster type. 'bulk_c' contains 1:1:1 booster, 'bulk_b' contains 'bulk' booster, 'bulk_b_e' contains bulk booster and empty wells. (**d**) Annotation of cells via FACS gating in FlowJo. BLAST = blasts, PROG = progenitors, LSC = leukemia stem cells. Source data are provided as a Source Data file.

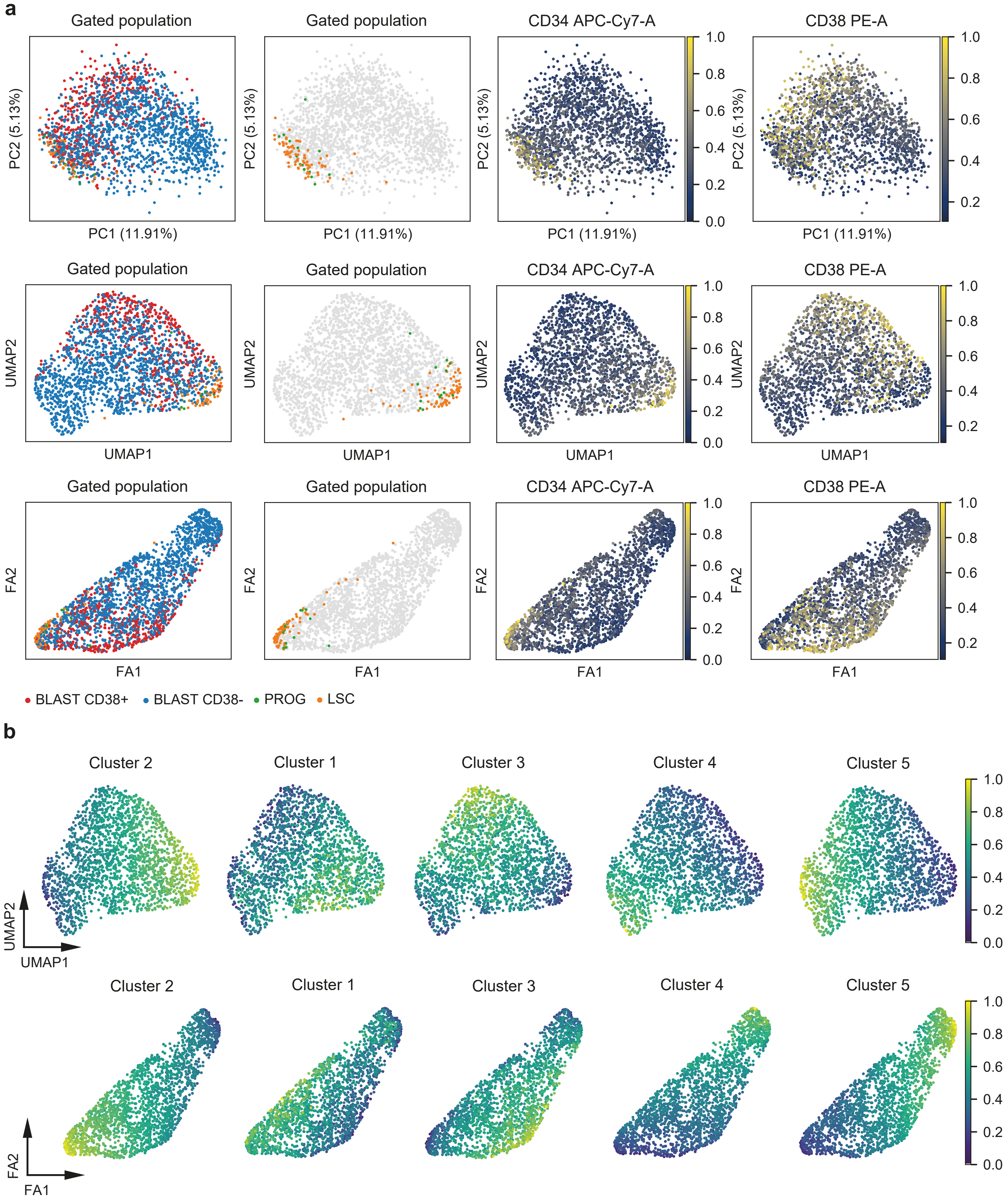

Supplementary Figure 8. Embedding of bulk dataset. (**a**) PCA, UMAP and Force-directed graph drawing (FA) of bulk dataset based on imputed scMS data (2,723 proteins, 2,025 cells) overlaid with FACS derived cell gating and CD34 and CD38 expression. BLAST = blasts, PROG = progenitors, LSC = leukemia stem cells. (**b**) UMAP and FA embedding overlaid with expression signature of clusters from Fig. 5. Source data are provided as a Source Data file.

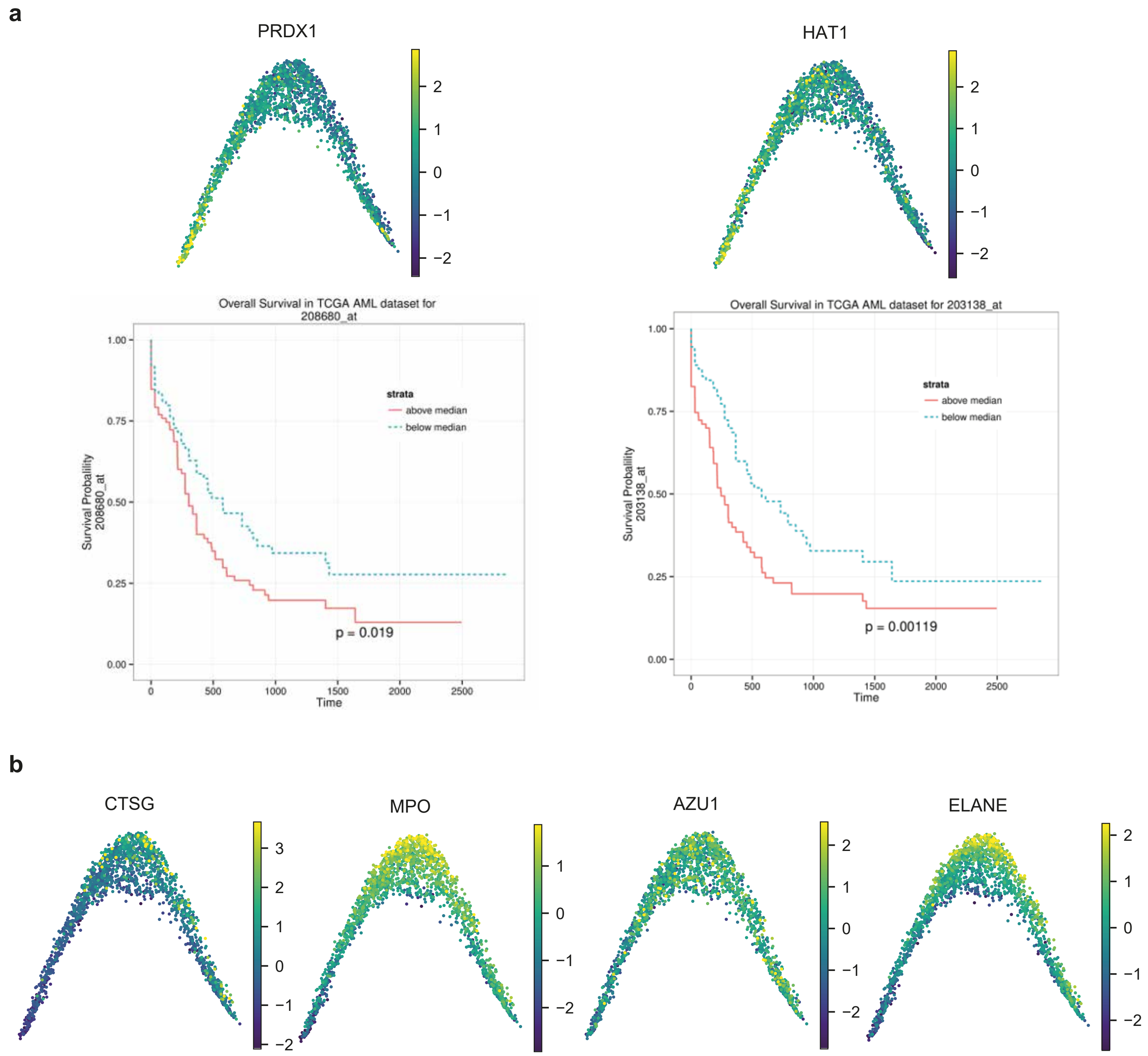

Supplementary Figure 9. Expression of selected proteins. (**a**) Top: Diffusion map based on imputed scMS data (2,723 proteins, 2,025 cells) overlaid with imputed and scaled expression of PRDX1 and HAT1. Bottom: Survival plots from bloodspot.eu, stratified by PRDX1 and HAT1 respectively. (**b**) Diffusion map based on imputed scMS data (2,723 proteins, 2,025 cells) overlaid with imputed and scaled expression of selected proteins from cluster 3. Source data are provided as a Source Data file.

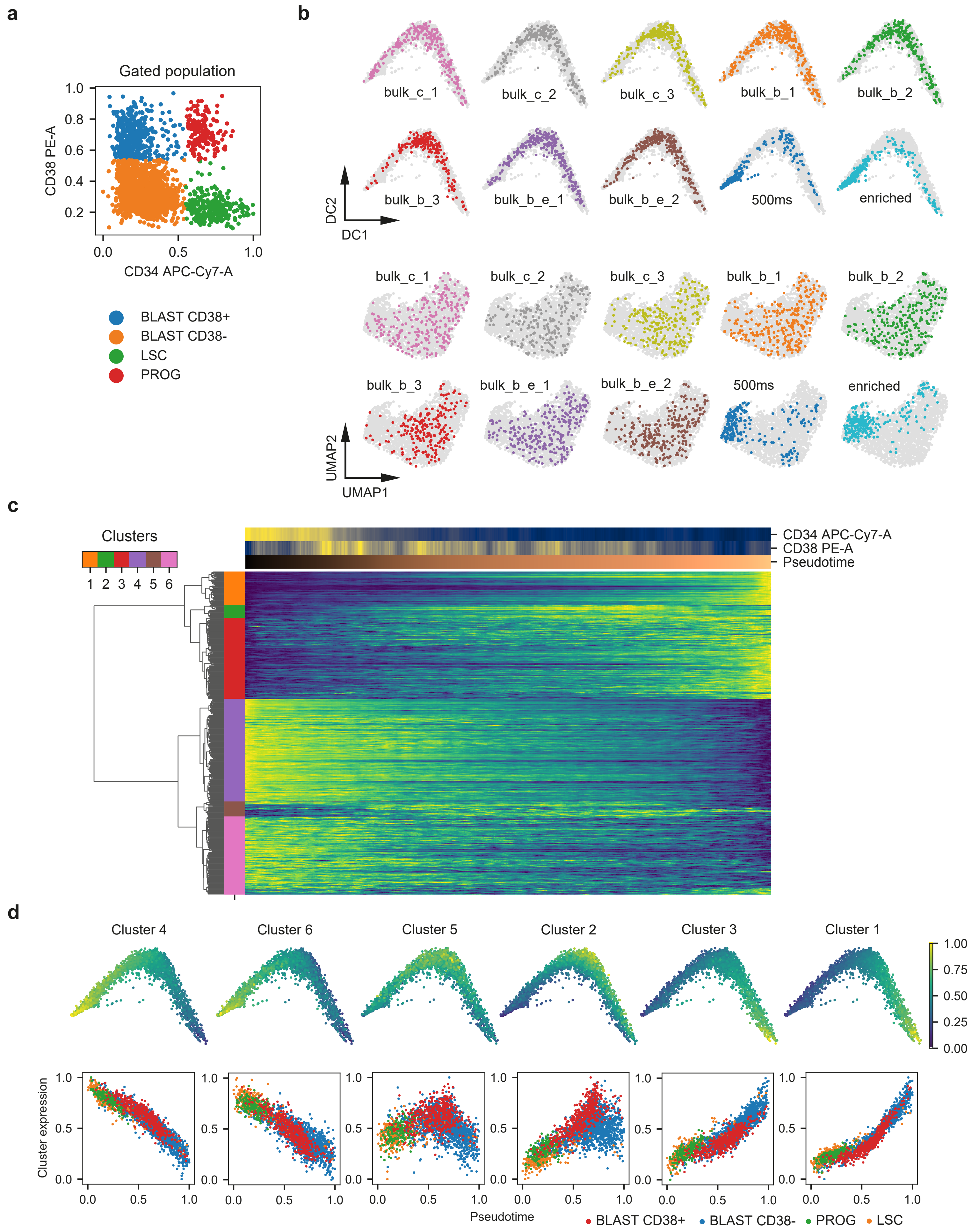

Supplementary Figure 10. Analysis of integrated dataset. (**a**) Scatterplot of 2,025 cells with FACS derived CD34 and CD38 expression. (**b**) Diffusion map and UMAP embedding of integrated dataset (2514 cells, 917 proteins), stratified by 384-plate. (**c**) Heatmap of cells in the columns ordered in pseudotime and 481 selected proteins (Methods) in the rows. Proteins were clustered hierarchically and clustered into five clusters. Protein expression values, CD34, CD38 and pseudotime for the ordered cells were smoothed by applying a moving average across 50 cells. Protein expression is normalized between 0 and 1. (**d**) Expression values of all proteins in each cluster were aggregated to a signature by taking the mean and normalizing between 0 and 1. Top: Signatures are plotted on top of the diffusion map. Bottom: Scatterplot of cells with their pseudotime and the signature of each cluster, annotated with their gating. BLAST = blasts, PROG = progenitors, LSC = leukemia stem cells. Source data are provided as a Source Data file.

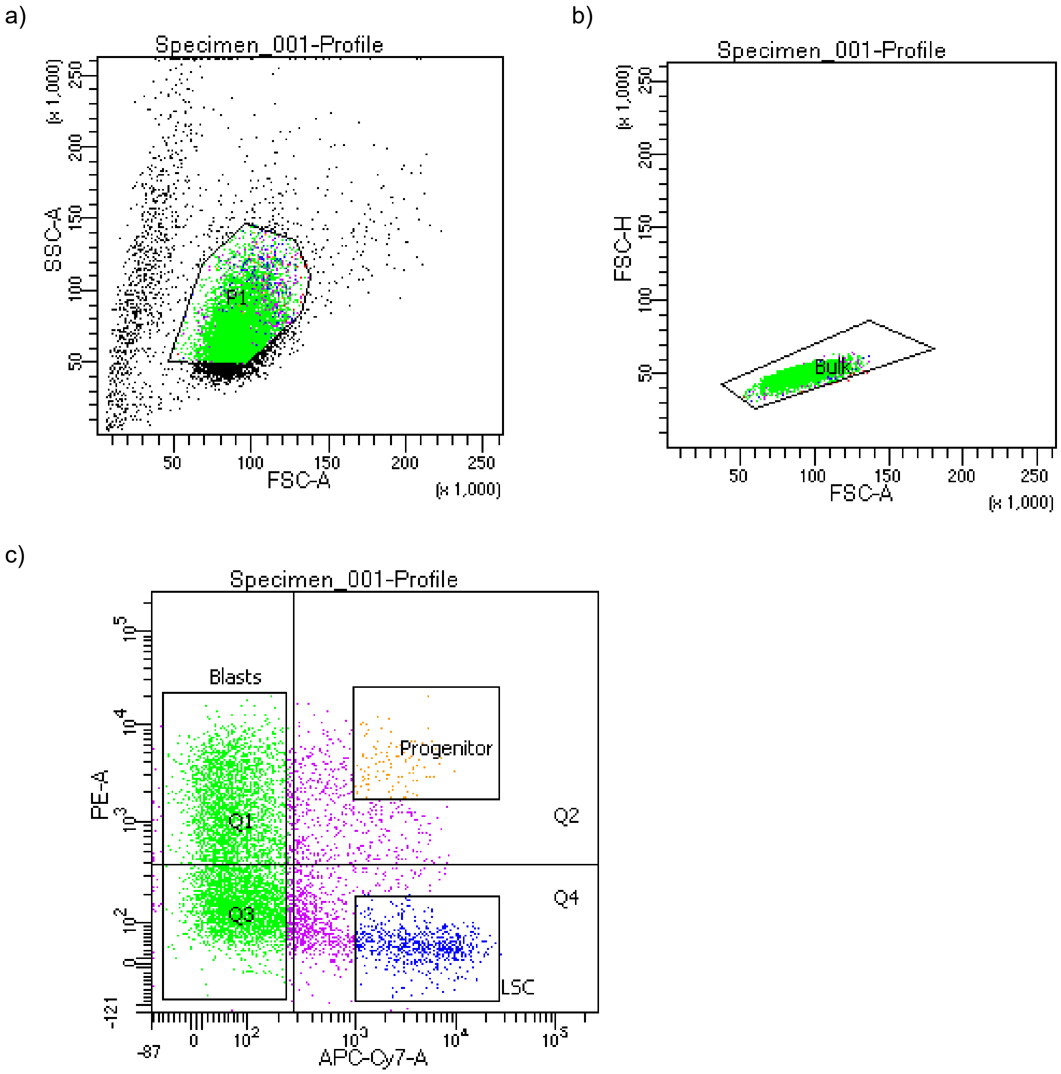

d)

Tube: Profile

Population	#Events	%Parent	%Total
All Events	10,000	####	100.0
P1	7,495	75.0	75.0
Bulk	7,474	99.7	74.7
Q1	2,605	34.9	26.0
Q2	566	7.6	5.7
Q3	2,997	40.1	30.0
Q4	1,306	17.5	13.1
LSC	642	8.6	6.4
Progenitor	103	1.4	1.0
Blasts	5,373	71.9	53.7

Supplementary Figure 11. FACS overview of the single cell sort of OCI-AML8227 cells, separating cells into blast, progenitor and LSC phenotypes. (**a**) P1 displaying gating of deemed live cells, (**b**) P2 of singlets, (**c**) blast, progenitor and Leukemia Stem Cell (LSC) gates. (**d**) Statistical overview of the various gates and cell frequencies. Source data are provided as a Source Data file.

Supplementary Table 1. Datasets generated in this study. 1:1:1 booster is an equimolar mix of blast, progenitor and LSCs, whereas Bulk booster represents live cells from the general OCI-8227AML culture system as a whole.

**Dataset**	**LC-MS method**	**# plates**	**booster**	**reference channel**	**empty channel**	**# single-cell channels**	**single-cell layout**
**300ms / medium**	Medium	1	1:1:1	-	-	14	5 LSC, 5 PROG, 4 BLAST
**500ms / high**	High	1	1:1:1	-	-	14	5 LSC, 5 PROG, 4 BLAST
**bulk_c**	High	3	1:1:1	X	-	13	Bulk
**bulk_b**	High	3	Bulk	X	-	13	Bulk
**bulk_b_e**	High	2	Bulk	X	X	12	Bulk
**enriched**	High	1	Bulk	X	-	13	4 LSC, 5 PROG, 4 BLAST

Supplementary Table 2. Parameters of the bulk single cell dataset.

	**bulk**
**Protein IDs**	2,870
**Peptide IDs**	17,158
**PSMs**	821,164
**PSM Rate [%]**	29
**Median s/n in Single-Cell Channels**	5.7
**Mean Protein IDs per file**	1,215
**Filtered Cells**	2025
**Filtered Proteins**	2,723
**Mean Protein IDs per Cell**	987
**Missing Values [%]**	63.75
