# Supplementary material for: Quantitative single-cell proteomics as a tool to characterize cellular hierarchies
Source: Nat Commun. 2021 Jun 7;12:3341. doi: 10.1038/s41467-021-23667-y (PMC8185083; doi:10.1038/s41467-021-23667-y)
Supplement: Supplementary file 3 — Description of Additional Supplementary Files [file 41467_2021_23667_MOESM3_ESM.docx]

**Description of Additional Supplementary Files**

**File Name: Supplementary Data 1**

**Description:** Overview of the sorting and TMT labeling layouts of the 384-well plates for the ’medium‘ and ’high‘ datasets.

**File Name: Supplementary Data 2**

**Description:** Differential expression and GO term analysis of our scMS data.
