# Supplementary material for: Quantitative single-cell proteomics as a tool to characterize cellular hierarchies
Source: Nat Commun. 2021 Jun 7;12:3341. doi: 10.1038/s41467-021-23667-y (PMC8185083; doi:10.1038/s41467-021-23667-y)
Supplement: Supplementary file 6 — Reporting Summary [file 41467_2021_23667_MOESM6_ESM.pdf]

nature research

Corresponding author(s): Erwin M. Schoof, Bo T. Porse

Last updated by author(s): May 4, 2021

Reporting Summary

Nature Research wishes to improve the reproducibility of the work that we publish. This form provides structure for consistency and transparency in reporting. For further information on Nature Research policies, see our Editorial Policies and the Editorial Policy Checklist.

Statistics

For all statistical analyses, confirm that the following items are present in the figure legend, table legend, main text, or Methods section.

n/a	Confirmed	
☐	☒	The exact sample size (n) for each experimental group/condition, given as a discrete number and unit of measurement
☐	☒	A statement on whether measurements were taken from distinct samples or whether the same sample was measured repeatedly
☐	☒	The statistical test(s) used AND whether they are one- or two-sided<br>*Only common tests should be described solely by name; describe more complex techniques in the Methods section.*
☒	☐	A description of all covariates tested
☐	☒	A description of any assumptions or corrections, such as tests of normality and adjustment for multiple comparisons
☐	☒	A full description of the statistical parameters including central tendency (e.g. means) or other basic estimates (e.g. regression coefficient) AND variation (e.g. standard deviation) or associated estimates of uncertainty (e.g. confidence intervals)
☐	☒	For null hypothesis testing, the test statistic (e.g. F, t, r) with confidence intervals, effect sizes, degrees of freedom and P value noted<br>*Give P values as exact values whenever suitable.*
☒	☐	For Bayesian analysis, information on the choice of priors and Markov chain Monte Carlo settings
☒	☐	For hierarchical and complex designs, identification of the appropriate level for tests and full reporting of outcomes
☐	☒	Estimates of effect sizes (e.g. Cohen's d, Pearson's r), indicating how they were calculated

Our web collection on statistics for biologists contains articles on many of the points above.

Software and code

Policy information about availability of computer code

Data collection	FACS sorting and data collection was done in BD FACSdiva v.8.0.2. MS data acquisition was done using Tune 1.1 and 2.0, and in Xcalibur v.4.3. TMT MS3 bulk cell library data was generated using Tune 3.4.
Data analysis	Mass spectrometry data was analyzed using Proteome Discoverer 2.4. Subsequent data analysis was performed in SCeptre, which is built using python 3.7.9 and scanpy 1.6.1dev102+g8d9eec4c with its dependencies on a Linux subsystem on Windows 10. The complete analysis is submitted with the manuscript. FlowJo v. 10.7.1 was used for analyzing .fcs files.

For manuscripts utilizing custom algorithms or software that are central to the research but not yet described in published literature, software must be made available to editors and reviewers. We strongly encourage code deposition in a community repository (e.g. GitHub). See the Nature Research guidelines for submitting code & software for further information.

Data

Policy information about availability of data

All manuscripts must include a data availability statement. This statement should provide the following information, where applicable:

- Accession codes, unique identifiers, or web links for publicly available datasets
- A list of figures that have associated raw data
- A description of any restrictions on data availability

The mass spectrometry data and SCeptre code (including FACS data) have been deposited to the ProteomeXchange Consortium (http://proteomecentral.proteomexchange.org) via the PRIDE partner repository with the dataset identifier PXD020586. Data has been made publicly accessible. The homo sapiens protein database was obtained from uniprot.org (Swissprot with isoforms, downloaded on 07/11/2020).

Field-specific reporting

Please select the one below that is the best fit for your research. If you are not sure, read the appropriate sections before making your selection.

- [x] Life sciences
- [ ] Behavioural & social sciences
- [ ] Ecological, evolutionary & environmental sciences

For a reference copy of the document with all sections, see nature.com/documents/nr-reporting-summary-flat.pdf

Life sciences study design

All studies must disclose on these points even when the disclosure is negative.

Sample size	For technical method comparisons, samples were measured in technical triplicates to enable the calculation of a CV. For the comparison of the biological interpretability of the 'medium' and 'high' method, over 200 cells were measured to account for biological heterogeneity. In the bulk scMS experiments a sufficient amount of cells were measured to ensure that rare cell differentiation stages such as progenitors were also present.
Data exclusions	No data was excluded from the study
Replication	For technical method comparisons, samples were measured in technical triplicates to enable the calculation of a CV. For the comparison of the biological interpretability of the 'medium' and 'high' method, 24 samples were measured respectively, representing biological replicates. The bulk scMS experiments were performed on 192 samples (biological replicates) using cells from 3 different days of FACS sorting (and thus, different cell passage). Thus, these results were successfully replicated across all biological replicates.
Randomization	To avoid technical covariates, TMT labeling of the single-cells was randomized and the order of MS-acquisition of the samples on each plate was randomized. FACS sorting of single cells in a non-enriched fashion also occurs according to random sampling.
Blinding	Blinding was not relevant to our study as manual sample handling steps were minimized and all samples were subjected to the same workflow. In the bulk scMS experiments, we were not aware of the differentiation stage of the cells until after data acquisition.

Reporting for specific materials, systems and methods

We require information from authors about some types of materials, experimental systems and methods used in many studies. Here, indicate whether each material, system or method listed is relevant to your study. If you are not sure if a list item applies to your research, read the appropriate section before selecting a response.

Materials & experimental systems

n/a	Involved in the study	
[ ]	[x]	Antibodies
[ ]	[x]	Eukaryotic cell lines
[x]	[ ]	Palaeontology and archaeology
[x]	[ ]	Animals and other organisms
[x]	[ ]	Human research participants
[x]	[ ]	Clinical data
[x]	[ ]	Dual use research of concern

Methods

n/a	Involved in the study	
[x]	[ ]	ChIP-seq
[ ]	[x]	Flow cytometry
[x]	[ ]	MRI-based neuroimaging

Antibodies

Antibodies used	CD34 APC Cy7 Clone 581 - Biolegend Cat# 343514, Lot# B284482<br>CD38 PE Clone HB7 - BD Cat# 347687
Validation	Each lot of this antibody is quality control tested by immunofluorescent staining with flow cytometric analysis to confirm similar results in terms of the CD34/CD38 profile (population percentages) of our OCI-AML8227 benchmark cell culture system.

Eukaryotic cell lines

Policy information about cell lines

Cell line source(s)	OCI-AML8227 cells were obtained from the John Dick Lab at the Princess Margaret Cancer Centre, Toronto, Canada
Authentication	The cell culture system was not authenticated
Mycoplasma contamination	The primary cell culture system was not tested for mycoplasma contamination

Commonly misidentified lines (See ICLAC register)	No commonly misidentified cell lines were used in the study

Flow Cytometry

Plots

Confirm that:

☒ The axis labels state the marker and fluorochrome used (e.g. CD4-FITC).

☒ The axis scales are clearly visible. Include numbers along axes only for bottom left plot of group (a 'group' is an analysis of identical markers).

☒ All plots are contour plots with outliers or pseudocolor plots.

☒ A numerical value for number of cells or percentage (with statistics) is provided.

Methodology

Sample preparation	8227 cells were stained at 5e6 cells / ml in StemSpan SFEM II media, using CD34 at 1:100 (vol:vol) and CD38 at 1:50 (vol:vol) for 30 minutes on ice in the dark. Cells were washed with 5x volumes of media, and then washed 3x in ice-cold PBS. Cells were filtered through a 40um cell strainer before FACS sorting.
Instrument	FACS Aria III
Software	FACSdiva 8.0.2
Cell population abundance	As the FACS instrument was run in single-cell purity mode, all sorted cells were subject to stringent filtering criteria by the instrument prior to depositing. Through index-sorting, we control for the actual immunophenotypic markers on the cells used in this analysis
Gating strategy	From FSC/SSC plots, live cells can easily be distinguished. Next, single cells are selected from . From the CD34 vs CD38 plot, previous functional characterizations (Lechman et al., Cancer Cell 2016) it is known that Blasts are CD34 negative, Progenitors are CD34+CD38+ and LSC are CD34+CD38-. From the statistics panel, it can be seen that selected Blasts occur at a 72% frequency, LSC at a 8.6% frequency and Progenitors at a 1.4% frequency.

☒ Tick this box to confirm that a figure exemplifying the gating strategy is provided in the Supplementary Information.
